# Cor Triatriatum Sinister: An Unusual Cause of Atrial Fibrillation in Adults

**DOI:** 10.1155/2018/9242519

**Published:** 2018-03-31

**Authors:** Christopher Hayes, Shuangbo Liu, James W. Tam, Malek Kass

**Affiliations:** ^1^Section of Cardiology, University of Manitoba, Winnipeg, MB, Canada; ^2^Section of Cardiology, University of Toronto, Toronto, ON, Canada; ^3^Cardiac Sciences Program, St. Boniface Hospital, Winnipeg, MB, Canada

## Abstract

Cor triatriatum is a rare congenital heart defect that is associated with an increased risk for developing atrial fibrillation. We report a case of a healthy 38-year-old man who presented in decompensated heart failure and atrial fibrillation with a rapid ventricular response. A transthoracic echocardiogram (TTE) demonstrated severe biventricular dysfunction and dilatation in addition to cor triatriatum sinister. He was diuresed with resolution of his symptoms and spontaneously converted back to sinus rhythm. There is limited evidence in the literature surrounding anticoagulation and associated left ventricular dysfunction in the setting of cor triatriatum which posed difficult therapeutic decisions.

## 1. Introduction

Cor triatriatum sinister is a rare congenital defect in which the left atrium is divided into two chambers by a membrane. Cor triatriatum comprises approximately 0.4% of congenital heart disease at autopsy [1]. It is found in less than 0.1% of clinically diagnosed cardiomyopathies [2]. It is typically identified in children and is a particularly rare new diagnosis in adults [3]. Atrial fibrillation has been described in 30% of published cases on adults with cor triatriatum [4]. We describe a case of an adult male who had known cor triatriatum (but not being actively followed) who presented in congestive heart failure in the setting of newly diagnosed atrial fibrillation and dilated cardiomyopathy.

## 2. Case

**Figure 1:**
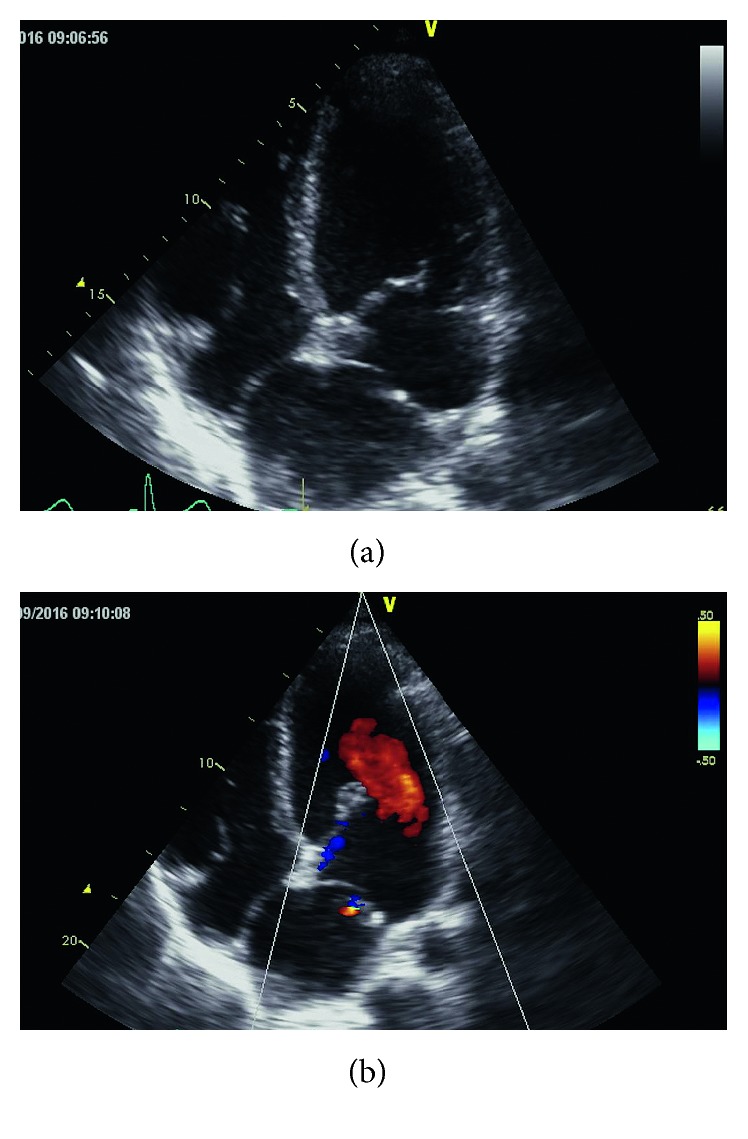
(a) An apical four-chamber view demonstrating a markedly dilated left atrium separated into two chambers by a membrane. (b) Colour Doppler images demonstrating flow across the fenestration in the membrane and across the mitral valve.

**Figure 2:**
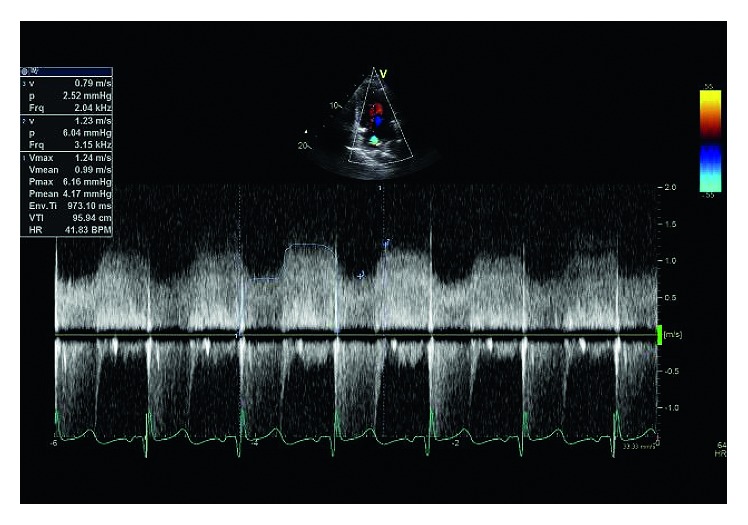
Continuous wave Doppler signal across the opening of the cor triatriatum membrane showing mildly accelerated flow throughout the cardiac cycle. The peak diastolic gradient was measured at 6 mmHg.

A previously healthy 38-year-old man, who had emigrated from Honduras 5 years ago, presented with a several weeks history of progressive abdominal pain and dyspnea. Physical examination revealed respiratory distress, an irregularly irregular tachycardia at 140 beats per minute, and a blood pressure of 126/72 mmHg. The cardiac exam was remarkable for a displaced and diffuse apical beat, and a 2/6 mitral regurgitation murmur heard at the apex. His ECG demonstrated atrial fibrillation (AF) with a rapid ventricular response. There was no electrocardiographic evidence of right ventricular overload. Chest X-ray showed cardiomegaly and pulmonary edema. Transthoracic echocardiography (TTE) demonstrated a markedly dilated left atrium in the four-chamber view (51 mm) divided into 2 chambers by a membrane (Figure 1). Continuous wave Doppler showed flow across the membrane with a peak diastolic gradient of 6 mmHg, peak systolic gradient of 2 mmHg, and a mean of 4 mmHg (Figure 2). The left ventricle (LV) was dilated in the parasternal long-axis view (65 mm) with an ejection fraction of 20–30%. There was moderate-to-severe functional mitral regurgitation. The right ventricular systolic pressure (RVSP) was 70 mmHg. Transesophageal echocardiography demonstrated a membrane within the left atrium (LA) with a 7 mm gap in its midportion and measured a peak gradient of 15 mmHg. The interatrial septum was intact. Of note, the patient had a previous TTE 5 years ago when first immigrating to Canada, which identified a dilated left atrium and the cor triatriatum defect which had a peak gradient of 9 mmHg, which was not that much different than the more current study. The LV was normal in size and systolic function at that time. The patient and his primary care provider were unaware of this prior established diagnosis.

The patient was admitted and diuresed. He spontaneously converted to sinus rhythm and remained free of AF at discharge. Given the coexistence of AF and cor triatriatum sinister, the decision was made to initiate anticoagulation with heparin with subsequent conversion to warfarin. The workup of alternative causes of his dilated cardiomyopathy (including serology for Chagas and autoimmune disease) proved to be negative. In addition, there was no history of significant alcohol intake. At 3 months follow-up, he remained in sinus rhythm and had returned to functional class 1 despite lack of improvement in LV systolic function on focused cardiac ultrasound evaluation.

## 3. Discussion

Cor triatriatum sinister is a rare congenital malformation involving an abnormal membrane dividing the left atrium into two chambers. It is thought to arise from failure to resorb components of the common pulmonary vein during embryogenesis [4]. The most common comorbid cardiac conditions in adults are atrial septal defects and mitral regurgitation [3]. Patients typically present with pulmonary edema in infancy unless a sizeable opening in the membrane allows for sufficient drainage of the affected pulmonary veins [3]. This opening may become obstructed later in life secondary to fibrosis and calcification and can lead to the development of symptoms [4]. Left atrial dilatation ensues from elevated filling pressures, and this substrate can give rise to the development of AF in a manner analogous to mitral stenosis [5].

Given the rarity of the diagnosis, formal guidelines do not exist on the optimal timing of surgical correction. Surgery has typically been offered in symptomatic adults [5]. In the largest surgical case series published, the mean age at the time of surgery was 27 years and the mean gradient across the membrane in those surgically repaired was 17.2 mmHg [5]. Outcomes after surgical repair have typically been excellent [5].

This case posed several unique diagnostic and therapeutic challenges. The etiology of dilated cardiomyopathy in this gentleman was not entirely clear. The initial suspicion was that the cardiomyopathy was secondary to uncontrolled tachycardia. This was challenged by the persistently depressed ejection fraction at 3 months follow-up despite apparent maintenance of sinus rhythm, and therapeutic doses of a beta blocker and ACE inhibitor. The workup for alternative etiologies had been negative, and no additional cardiac congenital abnormalities were discovered. Cor triatriatum has rarely been described in association with left ventricular failure, but in the setting of an additional insult such as pregnancy [6]. There is no reported association between cor triatriatum and idiopathic dilated cardiomyopathy. Apart from the development of atrial tachyarrhythmias and associated cardiomyopathy, there does not appear to be a known link between cor triatriatum and the development of dilated cardiomyopathy.

Cardioembolic stroke has been reported to occur with cor triatriatum with and without concurrent atrial fibrillation [7], and it has been proposed that clot formation in the left atrium shares a similar pathophysiology to mitral stenosis [8]. There are no data with regard to prophylactic anticoagulation in the absence of atrial fibrillation. Anticoagulation in this case was clearly indicated, given atrial fibrillation with the additional risk factor of congestive heart failure. Given the proposed left atrial pathophysiologic similarities with mitral stenosis, the decision was made to treat with a vitamin K antagonist.

Cor triatriatum is a rare but increasingly recognized congenital abnormality that can become symptomatic later in life. It is associated with the development of atrial fibrillation, pulmonary hypertension, and rarely with the development of left ventricular dysfunction. As access to imaging modalities and the quality thereof improve, more data will be needed to guide treatment decisions around thromboembolic prophylaxis and follow-up to prevent complications.
